# Community detection in directed weighted networks using Voronoi partitioning

**DOI:** 10.1038/s41598-024-58624-4

**Published:** 2024-04-07

**Authors:** Botond Molnár, Ildikó-Beáta Márton, Szabolcs Horvát, Mária Ercsey-Ravasz

**Affiliations:** 1 Faculty of Mathematics and Computer Science, Babeș-Bolyai University, 400084 Cluj-Napoca, Romania; 2 Faculty of Physics, Babeș-Bolyai University, 400084 Cluj-Napoca, Romania; 3 Transylvanian Institute of Neuroscience, 400191 Cluj-Napoca, Romania; 4 Department of Computer Science, Reykjavik University, 102 Reykjavík, Iceland; 5 Max Planck Institute for Cell Biology and Genetics, 01307 Dresden, Germany; 6 Center for Systems Biology Dresden, 01307 Dresden, Germany

**Keywords:** Complex networks, Computational science

## Abstract

Community detection is a ubiquitous problem in applied network analysis, however efficient techniques do not yet exist for all types of network data. Directed and weighted networks are an example, where the different information encoded by link weights and the possibly high graph density can cause difficulties for some approaches. Here we present an algorithm based on Voronoi partitioning generalized to deal with directed weighted networks. As an added benefit, this method can directly employ edge weights that represent lengths, in contrast to algorithms that operate with connection strengths, requiring ad-hoc transformations of length data. We demonstrate the method on inter-areal brain connectivity, air transportation networks, and several social networks. We compare the performance with several other well-known algorithms, applying them on a set of randomly generated benchmark networks. The algorithm can handle dense graphs where weights are the main factor determining communities. The hierarchical structure of networks can also be detected, as shown for the brain. Its time efficiency is comparable or even outperforms some of the state-of-the-art algorithms, the part with the highest time-complexity being Dijkstra’s shortest paths algorithm (${\mathcal {O}}(|E| + |V|\log |V|)$).

## Introduction

Many systems studied in social sciences, biology, neuroscience, information technology, etc. possess a connectivity structure and can be modelled as complex networks. Detecting communities—tightly connected groups of network nodes—is a common need in these fields. The connections in such networks may have directionality, and may also have attributes such as a *strength* or *length* or even both, which must be taken into account when performing community detection. These connection weights are particularly important in dense networks, where the network topology (i.e. the mere absence or presence of connections) may not carry as much information as the weights.

*Related works.* Many community detection algorithms were developed for undirected and unweighted networks^1–9^. Some support weights as well, but it is only a minority which handle networks in which links have both weights *and* directionality. The best known such algorithms are: 1) The Infomap method^7^, based on minimizing the so-called map equation (a cost function) with a stochastic approach, is usually well-performing and time efficient; 2) Stochastic block modelling^10^ also works well, however is computationally more costly; 3) Approaches that directly maximize the modularity measure, such as the Louvain^3^ and Leiden^11^ algorithms, can also be applied to directed weighted networks by using the directed generalization of modularity^12^. However, there are few practical and performant implementations that support this use case, a well known example being the leidenalg package^13^. While other heuristic algorithms have been published as well^14–17^, there are no easily accessible implementations for most of these, making it difficult to test them.

Here, we present a novel community detection method applicable to weighted directed networks based on the Voronoi partitioning of network nodes^8^. In this approach, a connection length is defined based on both the weight and the local topology of links, which allows for computing pairwise distances between the nodes in both directions. The generator points of the Voronoi cells are selected based on a local version of the *relative density* measure introduced in^1^, and an interpretable parameter *R*, called *radius*, which tunes the scale (or resolution) at which the communities are detected, it can indirectly control the number of communities that are found. Since partitionings obtained by this method are controlled by a single scalar parameter, *R*, it is straightforward to select optimal values of this parameter based on arbitrary quality measures (e.g. modularity). The method also allows for studying the hierarchical structure of the network, if it has one. The Voronoi community detection method is particularly advantageous when studying networks in which a natural connection length measure exists. Most other methods, such as those based on the concept of modularity, require transforming these *lengths* into a *strength* measure first, which is often done in an ad-hoc manner. This feature of the algorithm is comparable to how centrality measures like closeness or betweenness operate directly with connection lengths, in contrast to eigenvector centrality or PageRank which employ strengths instead. Another important feature of the algorithm is that communities obtained using Voronoi partitioning are contiguous, a desirable property that does not hold for all methods, especially naive modularity maximization ones such as Louvain^11^.

The method presented here is a generalization of an earlier Voronoi approach developed for undirected unweighted graphs^8^. The motivation for considering link weights and directions is not only that using more information has the potential of giving improved results, but also that there are examples of datasets on which the original algorithm could not provide any answer. For example inter-areal brain networks tend to be so dense that taking the symmetric (undirected) connectivity matrix and ignoring weights would result in an almost fully connected binary graph where clustering is meaningless. This new algorithm was developed to be general and flexible. Depending on the data, and the interpretation of link weights, one can choose an appropriate transformation to convert connection weights into connection lengths, to be used by the Voronoi algorithm.

We demonstrate applications of the algorithm on several real-world networks, namely: The weighted and directed brain connectivity network of the macaque and the mouse, obtained by retrograde-tracing experiments^18,19^, the weights are related to the probability of information transfer. As a second example, the algorithm was used to find communities in two social network datasets^20,21^, and third we applied the method on two geographical networks, a network of internal migrations between municipalities in Austria^22^ and a passenger air transportation network. In this last example, the geographical distances between airports were also taken into account, as an illustration of the flexible way in which the Voronoi algorithm can employ distance data. Finally, we evaluated the algorithm’s performance against other methods on a random benchmark set created using a weighted extension of the well-known Lancichinetti–Fortunato–Radicchi (LFR) network generator^23^ (weights representing the strength of connections). Comparisons were made with Infomap^7^, Louvain^3^, Leiden^13^ and stochastic block modelling algorithms^10^.

## Basic concepts

*Graph Voronoi diagrams.* While most frequently Voronoi diagrams are defined in Euclidean or other metric spaces^24^, they can also be defined on graphs^8,25^. Let $G=(V, E)$ be a weighted directed graph consisting of set *V* containing $N = |V|$ vertices/nodes and of set *E* containing $M = |E|$ edges/links. Let us denote the weight of the link $i\rightarrow j$ by $w_{ij}$. In order to define Voronoi diagrams one must introduce the concept of length for each edge $i\rightarrow j$: $l_{ij}>0$. In directed graphs $l_{ij}$ and $l_{ji}$ are not necessarily equal. Depending on the graph the length of edges may depend on the weight $w_{ij}$ of links given in the dataset. As usual, the length of a path is obtained by summing up the lengths of edges along it. The distance *d*(*i*, *j*) will denote the length of the shortest path going *from* node *i* *to* node *j*. Here we focus on weighted directed graphs.

Let us choose a set of generator points (seeds) $S\equiv \{\gamma _1, \gamma _2, ..., \gamma _g\} \subset V$. We define the Voronoi diagram of *G* with respect to *S* as a partitioning of *V* into disjoint subsets $V_1, V_2,..., V_g \subset V$ called *Voronoi cells*, where each cell is associated with a generator point, and they have the following two properties: (1) the Voronoi cells cover the original graph with no overlaps: $\cup _{i=1}^{g}V_i=V$ and $V_i\cap V_j=\emptyset$, $\forall i \ne j$; (2) nodes in a Voronoi cell are closest to the generator point of that particular cell: $d(n,\gamma _i)\le d(n,\gamma _j)$, $\forall n \in V_i, i,j=1, 2, ..., g$. If, by a small chance, a node is at equal distance from two seeds we can choose its cell randomly between the two.

## The algorithm

When defining our community detection algorithm based on Voronoi diagrams, we need to choose a distance measure between nodes that preferably accounts both for topology and link weights, and even distances if they are available in some datasets. We also need to define a way of choosing generator nodes.

*Distance measure*. While in unweighted networks one needs to consider only the topology of the network, here weights have a strong effect on community formation, posing also a greater challenge for detecting communities. We define the length $l_{ij}$ of link $i \rightarrow j$ based on two features of the network data:

(1) Topological effects, i.e. the actual connectivity structure, incorporated by calculating the edge clustering coefficient $C_{n_i, n_j}$ (ECC^26^):

1 $$\begin{aligned} C_{n_i, n_j}=\frac{z(n_i, n_j) + 1}{\min [k(n_i) - 1, k(n_j) - 1]}, \end{aligned}$$

where $z(n_i, n_j)$ denotes the number of common neighbors of nodes $n_i$ and $n_j$, i.e. the number of triangles in which the $(n_i, n_j)$ edge participates, while $k(n_i)$ and $k(n_j)$ are the degrees of $n_i$ and $n_j$. Edge weights and edge directions are not taken into consideration when computing this measure. For defining the length of the link we take $l_{ij}\sim 1/C_{n_i,n_j}$^8^.

(2) The best way to take edge weights into account depends on the specific dataset and the interpretation of weights: do they represent a kind of connection strength, correlation measures, physical distances, bandwidth, flow measures, etc.? For now let us write the relationship between the length $l_{ij}$ of a link and its weight $w_{ij}$ in a general form, as $l_{ij}\sim f(w_{ij})$. The proposed length measure in this paper is a combination of the above mentioned two measures:

2 $$\begin{aligned} l_{ij}=\frac{f( w_{ij})}{C_{n_i, n_j} }. \end{aligned}$$

The appropriate form of the $f(w_{ij})$ transformation depends on the dataset and needs to be determined by the user of the algorithm. The main possibilities are the following.

1. In some datasets, weights $w_{ij} \in (0,1]$ represent the probability of information flow along a given out-link of a node. A first approximation for this probability can often be obtained from correlation measures, normalized bandwidth of information transfer, or other measures. The probability for the information to travel along $n_i \rightarrow n_j \rightarrow n_k$ is proportional to $w_{ij} w_{jk}$. Then the logarithmic form of weights, $-\ln w_{ij} > 0$, is additive, and the shortest paths indicate routes where information flows with highest probability. This has been used in case of structural^27^ and functional brain networks^28^, state transition networks^29^, etc. In such situations we choose $f(w) = -\ln |w|$. In case of weights representing correlation measures the absolute value of the weights must be considered, because strong negative correlation also indicates strong information transfer, although such choice always depends on the data set itself and must be made on a case-by-case basis. We illustrate this method on the inter-areal cortical brain network of the macaque monkey^18^ and the mouse^19^ (see Section *Structural brain networks*), and also on the Austrian migration network^22^ (see *Geographical networks*).
2. In many cases weights represent some type of connection strength and it is desired that the algorithm should generally place nodes connected by high-weight links into the same community. Therefore, one needs to choose a transformation that converts large weights into short lengths. A straightforward choice is $f(w) = 1/w$. In the current study, this methodology is used on a set of social networks (see Section *Social networks*) and on a large set of benchmark networks with various degree distributions, inter- and intra-community weight distributions (see Section *Evaluation against other algorithms on artificial benchmark networks*).
3. In some special cases the nodes are embedded in physical space and their geometric distances, $d_{ij}$, are already known. Besides these we might have other information for weight measures. In these cases there are several possibilities how to take into account all the information. We chose to illustrate this on an air transportation network, where we present 4 different choices (see Section *Geographical networks*), all giving reasonable results.

**Figure 1:**
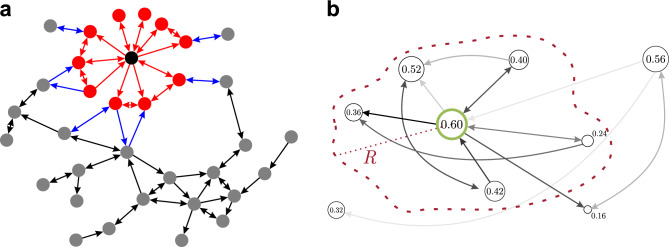
Sketch of the algorithm. (**a**) In order to calculate the weighted local node density $\rho _i$, we first count the links inside the first order neighborhood (red, $m=24$ in this example) and the links outgoing from and incoming into the same neighborhood (blue, $k=12$ in this example). Links between two first order neighbors are also considered inside links (red). The unweighted local relative density would be $m/(m+k) = 2/3$, this needs to be multiplied by the weighted degree of node *i*. (**b**) The size of nodes represents their weighted local relative density $\rho$, while the shade of links shows their weight *w*. Generator nodes (green border) are selected so that they have the highest weighted local relative density within a region of radius *R* around them (red dashed line). No other generator points can exist within this region.

*Selecting generator nodes.* The proper identification of the generator nodes is of key importance for obtaining Voronoi cells that are in good correlation with the community structure of the graph. The goal is to identify a generator point in each community such that the Voronoi cell induced by it would coincide with the community. It has been proposed in the past, both in the context of general clustering and community detection in networks, that clusters are typically concentrated around *density peaks*^30,31^. Therefore, we generalize the *local relative density* measure applied in^8^ to a weighted context as follows:

3 $$\begin{aligned} \rho _i=s_i\frac{m}{m+k}, \end{aligned}$$

where $s_i$ is its total (incoming and outgoing) weighted degree or *strength*, *m* is the number of edges within the first order neighborhood of node *i*, while *k* is the number of edges entering into or exiting the same neighborhood. Links between two first order neighbor nodes are considered as inside edges, as shown in Fig. 1a. We refer to the quantity $\rho _i$ as the weighted local relative density of node *i*.

Node *i* is chosen as generator point if it has the highest local relative density in a region within radius *R*: $\rho _i>\rho _j, \forall j\ne i , d(i,j)<R$. Algorithmically, to determine the generator points, first we select the node with the highest local relative density, then exclude all nodes from which it is no more than distance *R* away. Then repeat the procedure on the remaining nodes until the entire network has been covered. This requires as many single-source shortest path calculations as the final number of generator points, determined by *R*. When using an optimal implementation of Dijkstra’s algorithm^32^, the procedure takes $O\bigl (g(|E| + |V| \log |V|)\bigr )$ operations, where *g* denotes the number of generator points.

In networks that display a strong community structure, the inter-community node distances will be markedly smaller than the intra-community ones. This means that in order to detect communities accurately, it is important that each one should have exactly one generator point within it. However, the precise location of a generator point within its community does not influence the result significantly. We will demonstrate this on benchmark networks and the macaque brain network by randomizing the location of generator nodes (Section *Robustness of the generator point selection*).

In special data sets where some extra information is known about the importance of nodes, one may use this extra information to select the generator nodes. This adds extra flexibility to the algorithm. We demonstrate this using air travel data, using airport sizes to select generators (see Section *Geographical networks*).

*Node assignment to clusters.* In order to compute the Voronoi partitioning of a graph, one must find the closest generator point to each vertex (Fig. 1b). From an algorithmic perspective, the simplest way to do so is to pre-compute the distances between all pairs of vertices using the Floyd–Warshall algorithm^33,34^, which is simple to implement, but takes time proportional to $|V|^3$, where |*V*| represents the size of the vertex set of the graph. However, when the number of generator nodes is small, the calculation can be performed more efficiently using a single-source shortest path algorithm, applied once to each generator point. We used Dijkstra’s algorithm for this, implemented using a binary heap, which takes time proportional to $|S| \, |E| \log |V|$ for connected graphs, where |*S*| and |*E*| are the sizes of the generator set and the edge set of the graph, respectively. An additional optimization is possible by limiting the shortest path search from each generator up to those vertices which are not closer to any previously processed generator point. This way, the shortest path search will explore successively smaller and smaller regions of the graph with each new generator point, leading to a sub-linear complexity in the number |*S*| of generators. On average, we expect to explore a fraction 1/*k* of the graph when processing the *k*th generator, therefore the time to compute the entire Voronoi partitioning will be proportional to $\sum _{k=1}^{|S|} 1/k \sim \log |S|$, yielding a final computational complexity of $O(\log |S| \, |E| \log |V|)$. The logarithmic scaling in the number of generators allows us to efficiently handle a large number of generator points, which is necessary when maximizing quality metrics of the partitioning in terms of the radius parameter *R*.

*Selecting the optimal clustering.* In order to choose an optimal partitioning of the graph, one may vary the *R* scale parameter so as to maximize some quality measure. Here we chose the directed and weighted generalization of Newman modularity for this purpose^2,12^, defined as:

4 $$\begin{aligned} Q=\frac{1}{m}\sum _{i,j\in V}\left[ w_{ij}-\frac{s_i^\text {out}\cdot s_j^\text {in}}{m}\right] \cdot \delta _{c_i, c_j}, \end{aligned}$$

where $m = \sum _{i,j} w_{ij}$ denotes the total sum of edge weights in the graph, $s_i^\text {out}$ and $s_j^\text {in}$ represent the weighted out- and in-degree of nodes *i* and *j*, while $\delta _{c_i, c_j}$ is the Kronecker symbol, which equals to 1 if nodes *i* and *j* are in the same cluster, $c_i=c_j$, and 0 otherwise. Many studies argue^2,4,6,35^ that optimizing modularity is a good way of detecting community structure, however, it is not universal as shown by^3^. We must also emphasize that even if we choose an *R* value based on searching for high modularity, the partitioning itself does not directly optimize the modularity function as in the Leiden or Louvain algorithms^3,13^. The maximization in terms of the single *R* parameter may be carried out with any derivative-free optimization technique. We chose Brent’s method^36,37^ for its simplicity, bracketing the optimum between the graph diameter (largest possible *R* value) and smallest edge length (smallest *R* value).

## Empirical analysis

### Structural brain networks

First we demonstrate the clustering algorithm on anatomical brain connectivity data from the macaque^18^ and mouse brains^19^. In this dataset, connection weights represent the relative fraction of connections incoming from each brain area. Since these are interpretable as probabilities for information transfer along given links, we choose the transformation $f(w) = -\ln w$. The macaque cortex can be divided into 91 functional areas, out of which the connectivity of 29 areas was mapped in^18^, meaning that only a subnetwork of 29 nodes is completely known. This network has a density of $66\%$ with a log-normal weight distribution and it was shown that weights carry important information, being dependent also on the physical distance according to the so-called exponential distance rule^27^. The mouse dataset contains the connectivity of 23 areas (out of 47 in total), which form a highly dense graph ($96\%$ density).

**Figure 2:**
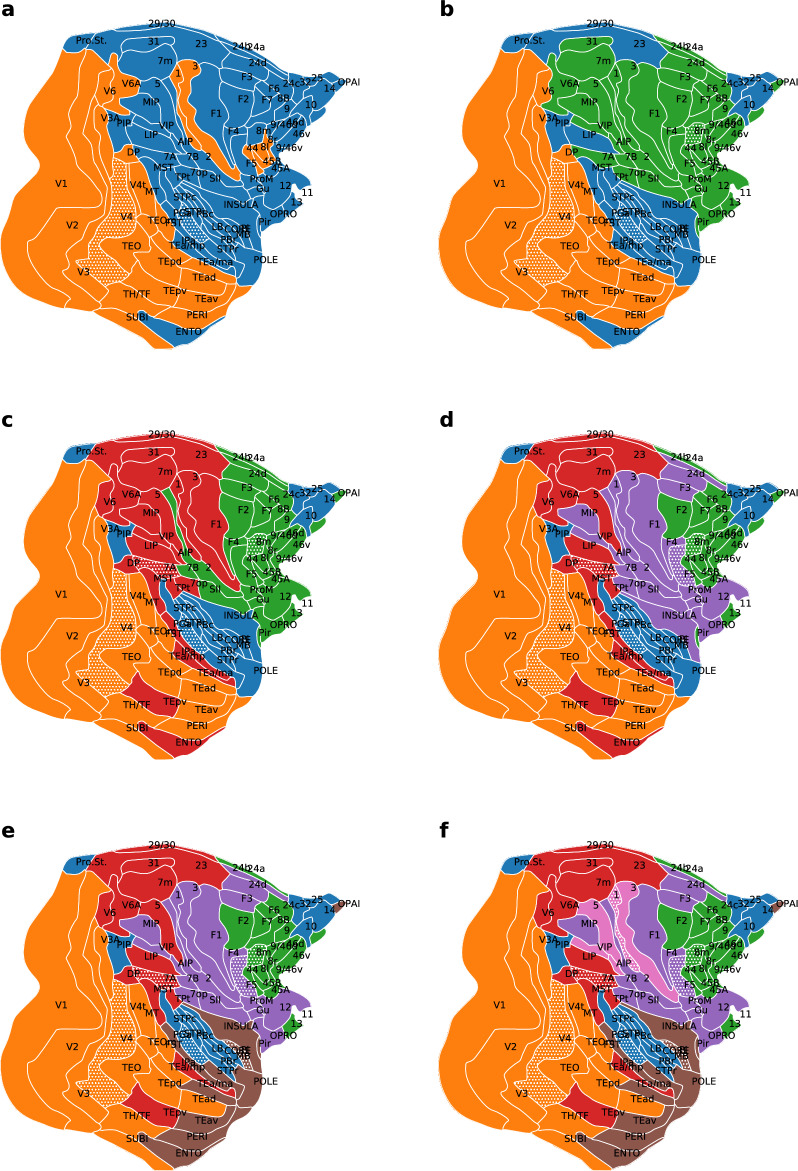
Clustering of cortical brain areas in the macaque based on their structural connectivity. Colors represent different clusters, while the generator nodes are marked with a dotted pattern. Connectivity data was completely known for 29 areas, for the remaining portion of the network connections were predicted using a ML algorithm^38^. Panels (**a**)–(**f**) show partitionings into 2–7 clusters, as obtained with the Voronoi algorithm. The highest modularity is achieved with 5 clusters (panel (**d**)).

**Figure 3:**
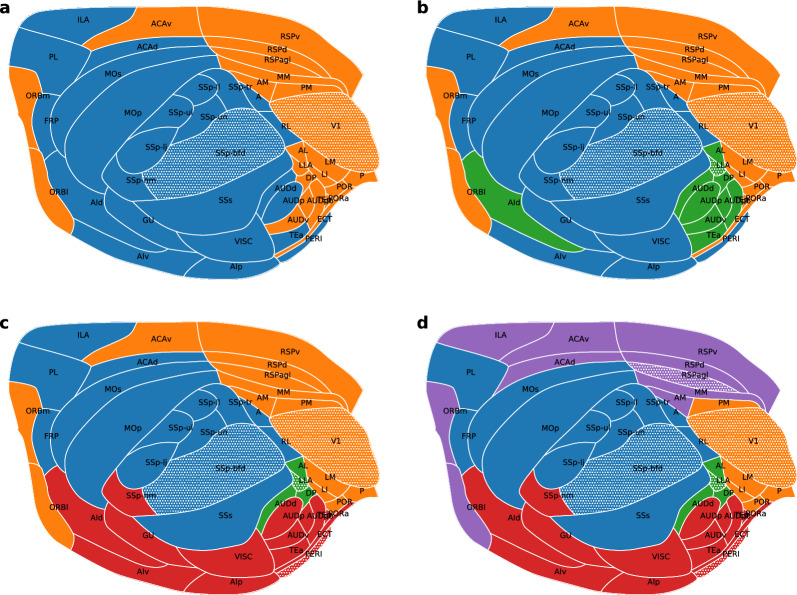
Clustering of the brain network in case of the mouse. Colors represent different clusters, the patterns show the generator nodes of clusters. The full connectivity network was obtained by predicting the connection weights using a ML algorithm, based on the fully known, experimentally determined connections of 23 areas. The Voronoi algorithm provided a fixed number of clusters, as follows: (**a**) two; (**b**) three; (**c**) four; respectively (**d**) five. In case of the mouse the optimal clustering is obtained with 4 clusters (panel (**c**)).

However, missing connections can influence the distribution of areas (nodes) into optimal clusters, thus the clustering was performed on the full network, where connections between areas were predicted using a machine learning algorithm. Imputation was performed between all area pairs, based on the completely known subgraph and the full distance matrix, generating samples for a complete inter-arial network for both monkey and mouse (details in^38^). Thus, in these networks, most information is carried by the connection weights. The Voronoi clustering algorithm yielded 5 clusters in the optimal state for the monkey (considering the highest Newman modularity) (Fig. 2d) shown on the 2D brain flatmap. In case of the mouse the optimal number of clusters proposed by the algorithm is 4 (Fig. 3c). In Figs. 2 and 3 colors represent different clusters and the ones with patterns are the generator points for the particular cluster. To form a more complete picture several fixed cluster number states were also generated by the algorithm for both animals, ranging from 2 clusters up to 7 in case of the monkey (Fig. 2a–f) and up to 5 clusters in case of the mouse (Fig. 3a–d). One can observe that by reducing the scale parameter *R*, large clusters break up into smaller ones, revealing the hierarchical structure of the brain. We emphasize that in these cases the physical distances between brain regions were not used—only the connection weights between areas were employed. However, we know that a direct relationship between weights and distances exists (the exponential distance rule, saying that the number of axons crossing the white matter decreases exponentially with length^27^). Indeed, looking at the brain flatmaps it is apparent that clusters are physically localized. Note that these being flatmaps of a three-dimensional brain, in some cases regions on opposite sides of the plot are physically near each other, for example see the orange areas in the mouse brain in Fig. 3.

In the monkey the two big clusters (orange and blue in Fig. 2a) roughly coincide with the dorsal and ventral stream in the brain separating mainly the upper and lower parts of the brain (see^39^). As the number of clusters grows areas with different functions get separated into different clusters. Looking at the optimal clusterization (Fig. 2d)) we still see the visual areas (mainly the ventral visual stream V1, V2, V3, V4, MT, TEO, etc.) in one big cluster (orange), the red cluster seems to be the dorsal visual stream. In case of having more detailed information about the large visual areas, V1 and V2 (braking them into smaller parts), one could probably draw even more interesting conclusions about the two-stream hypothesis^39^ and the visual information flow in the brain. The blue cluster contains mainly areas from the temporal lobe (STPr, STPi, STPc, PBr, etc.) but also includes some interesting long-range connections to the prefrontal lobe. The purple cluster includes mainly frontal areas, the green cluster mainly prefrontal areas with a few exceptions on the periphery of the two.

In the mouse brain the two large clusters have their generator nodes in V1 (the primary visual area), and SSp-bfd (the primary somatosensory area, barrel field), and these clusters separate relatively well the visual areas and somatosensory areas. When the third cluster appears this contains most auditive areas (green). All these observations indicate that the clustering method gives us biologically meaningful results.

To demonstrate how missing information can affect the optimal clustering, we also performed the partitioning of the original, incomplete cortical networks, obtaining 7 cluster optimal state in case of the monkey, respectively 2 clusters in case of the mouse. The obtained results are shown on the 2D flatmaps in the Supplementary Information (Figs. S1, S2). One can see that including the missing information the accuracy and the biological meaning of the clustering is improved.

### Social networks

**Figure 4:**
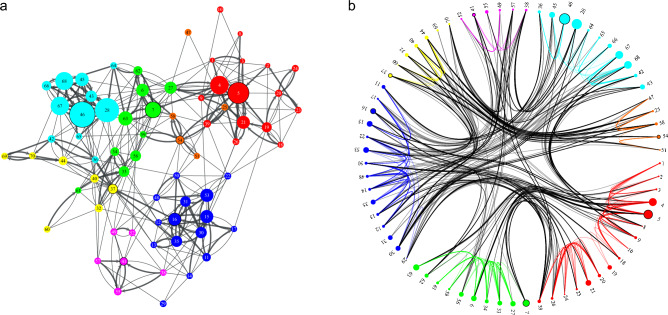
Clustering of a friendship network of high school students. A link is present between student *i* and student *j* if *i* named *j* as a friend, in either of the two identical surveys conducted, one from Fall and another in Spring semesters. Edge weights represent the number of surveys in which the friendship was named. Panel (**a**) shows the network using the coordinates present in the database, obtained using a force-based layout. Panel (**b**) shows the same network on a circular layout, with nodes ordered according to their cluster assignments. Colors indicate clusters. Generator nodes are highlighted with a black border. Link widths are proportional to weights.

**Figure 5:**
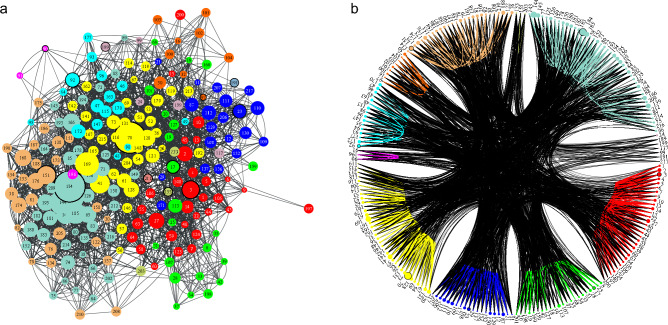
Clustering of a friendship network of university students sharing a residence hall. A link is present between student *i* and student *j* if *i* named *j* as a friend. Edge weights indicate the level of their friendship. Panel (**a**) shows network using the coordinates present in the database, obtained using a force-based layout. Panel (**b**) shows the same network on a circular layout, with nodes ordered according to their cluster assignments. Colors indicate clusters. Generator nodes are highlighted with a black border. Link widths are proportional to weights.

We tested our method also on a set of social networks obtained from the open Netzschleuder database^40^. First the method was applied to a network of friendships, where data was collected in a small high school in Illinois in 1958 among the male students. A weighted link represents how many times student *i* named student *j* as a friend, in either of two identical surveys conducted (one in Fall semester and one in Spring semester)^20^. A bigger and more complex social network, also obtained from the same database is the friendship network of students living in the same residence hall at the Australian National University. Edge direction indicates that resident *i* named resident *j* as a friend, and edge weight indicates the level of their friendship: 5 (best friend), 4 (close friend), 3 (friend), 2, 1.^21^. In these friendship networks link weights represent the strength of friendship, so when calculating the length of edges we applied the approach $f(w_{ij})=1/w_{ij}$. Results are shown in Figs. 4 and 5. We applied both a force-based layout algorithm and also a circle layout to illustrate the relation of the network structure to the communities obtained.

### Geographical networks

**Figure 6:**
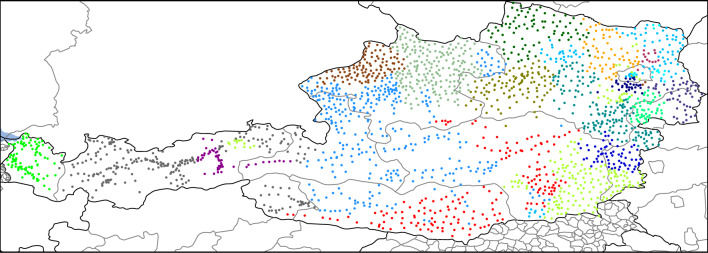
Communities in the network of Austrian internal migrations between municipalities. A weighted directed link represent the migration flow between municipalities. Each dot on the map represents an Austrian municipality. Colors indicate clusters, while borders on the Austrian map show different administrative regions. Links are not shown in this plot.

The next dataset we used represents internal migrations between municipalities in Austria between 2002 and 2023^22^. A weighted directed link from source municipality to target municipality indicates the migration flow between them. Edges are annotated with migration volume (number of people), nationality, sex, and year, however in this study we only used the volume of migration summed up over the entire covered time period. In this network we calculated links as $f(w_{ij})=-\ln w_{ij}$, where $w_{ij}$ represent normalized flows. This is analogous to the approach used for brain networks, which carry information flow. The result is shown in Fig. 6. The obtained communities clearly correlate with Austria’s administrative regions, demonstrating that the results are meaningful.

A second example of a geographic network is the air transportation network provided by the Bureau of Transportation Statistics of the United States^41^. The nodes of this network are airports and the links are airline routes. The database used in the current study is for the year 2018 and contains data for passenger counts between any two airports in the United States. The database also contains the great circle distance between the airports, which we denote by $d_{ij}$. To compensate for the incompleteness of the database, only the strongly connected giant component of the constructed network is used in this study, consisting of 1106 airports. We also have information about the largest airports^42^.

**Figure 7:**
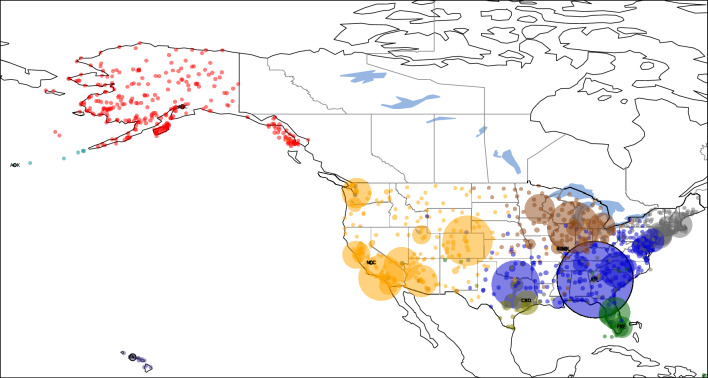
Clustering of the air passenger transportation network of the United States using the Voronoi algorithm, yielding 28 clusters, comprising of 8 large and 20 small clusters. In this approach weights are the total passanger count and the length of links is $l_{ij}=d_{ij}/C_{n_i, n_j}$. The disks represent the locations of airports on the map. Their radius is proportional to their local relative density, and their color indicates the cluster they belong to. Generator nodes are shown with a black border. Only generator point airports are annotated with the IATA airport code (ATL, HNL, ANC, NLC, CXO, BLV, ENL, FXE).

**Figure 8:**
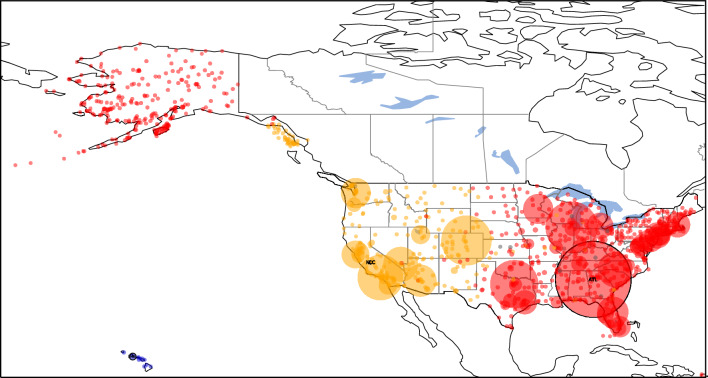
Clustering of the air passenger transportation network of the United States using the second approach, where weights are the total passanger count and $l_{ij}=d_{ij}$. The algorithm yields 10 clusters, comprising of 3 large and 7 small clusters. Generator node airport codes are as follows: ATL, HNL, NLC.

**Figure 9:**
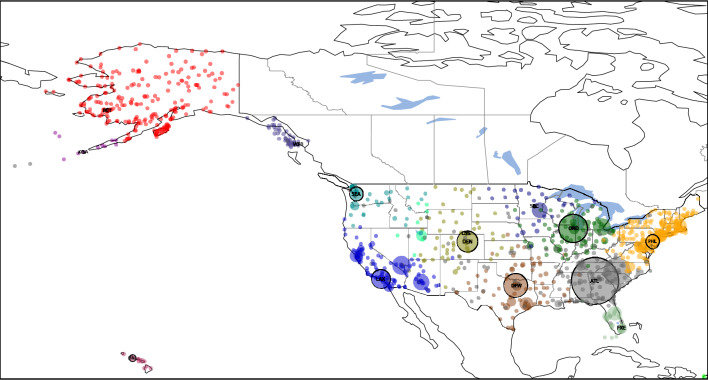
Clustering of the air passenger transportation network of the United States using the third approach, where the weight of links is the total passenger count normalized with distance and $l_{ij}=d_{ij}$. We obtain 116 clusters, comprising 14 large clusters and 102 small clusters, with generator points annotated using their IATA codes: ATL, ORD, DFW, DEN, LAX, PHL, SEA, HNL, FXE, STC, WFB, BET, KQA, CYS.

**Figure 10:**
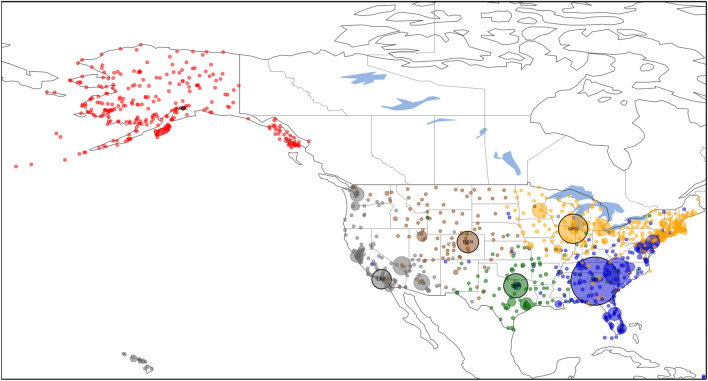
Clustering of the air passenger transportation network of the United States using the Voronoi algorithm using the Top 5 busiest airports (ATL, LAX, ORD, DFW, DEN) and ANC (Anchorage, Alaska) as generator points.

Here we demonstrate different approaches for defining weights, the length of links, and even the generator nodes. We present 4 possibilities:

1. The link weight is the passenger count and these weights are used to identify generator nodes. Length of links are defined using $f(d_{ij})=d_{ij}$ so $l_{ij}=d_{ij}/C_{n_i, n_j}$, the distance over the edge clustering coefficient. The result is shown in Fig. 7. The Voronoi clustering algorithm yields a total of 28 clusters, out of which 8 clusters are big communities, comprising the majority of the nodes; and 20 small clusters with less than 5 nodes in the community. Is it apparent that the clusters correspond to major geographical regions of the United States: East Coast (gray), Midwest (brown), Central around Atlanta (blue), West Coast (yellow), Alaska (light red), Florida (green) and Hawaii (purple). Also, the local relative density of the nodes is proportional to the annual traffic of the airport, defining Hartsfield–Jackson Atlanta International Airport (ATL) as the first generator point, it being the busiest airport in the United States.

2. Having a network embedded in physical space, we also try the version where the length of link is simply the physical distance: $l_{ij}=d_{ij}$. The weight of links is the passenger count as in case 1. Results are shown in Fig. 8. Using the Voronoi clustering algorithm the nodes of the network were distributed in a total of 10 clusters, out of which 3 clusters are big communities, comprising the majority of the nodes; and 7 small clusters with less than 5 nodes in the community. One can see that the 3 major clusters divide the United States in two major parts Central-East (red) centered around Atlanta International Airport (ATL) and Central-West (yellow). Hawaii also tend to form its own cluster (purple), with its generator airport being Honolulu International Airport (HNL). It is very interesting to see that Alaska has a strong connection to the Central-East cluster. Also, the local relative density of the nodes is proportional to the annual traffic of the airport, defining Atlanta International Airport (ATL) as the first generator point, it being the busiest airport in the United States.

3. There are several possibilities also for the definition of link weights. As a second option we take the edge weights $w_{ij}$ as the total yearly passenger count of the route divided by its geographic length, obtaining a value of dimension $\text {passenger}\, per \,\textrm{km}$. We argue that this normalization makes the weights more comparable to each other. It captures the intuition that if two routes of different lengths have the same passenger counts, then the shorter connection should be considered the *stronger* one. On short routes, one would generally expect smaller passenger counts since it is feasible to use other means of transport than air travel. The Voronoi algorithm yields an optimal clustering consisting of the 116 communities shown in Fig. 9. Of these, only 14 are large enough to be clearly visible in the figure, but there are also 102 tiny clusters, often consisting of a single node. These tiny clusters appear because the database is often incomplete (number of links, passenger counts etc.), especially in case of small airports, which often become their own isolated cluster. This drawback can be corrected by using a more complete database, which was not available at the time of this study. However, examining only the 14 large clusters one can conclude that the algorithm separates extremely well the airports of different major US geographical regions: the East Coast (orange), Midwest (light blue), West Coast (brown), Alaska (cyan), Southeast (light orange) and Central around Atlanta (blue) regions. Often, the selected generator points for these clusters are the major airport hubs of that particular region, e.g. Hartsfield-Jackson Atlanta International Airport (ATL), Chicago O’Hare International Airport (ORD), etc.

4. And finally, given that a key feature of the algorithm is its ability to handle predefined generator nodes, we also investigated the case where the generator nodes are identified based on the top lists of airports^42^. The length of links is still $l_{ij}=d_{ij}$. To show this, in Supplementary Figs. 3 and 4 the top 5, respectively the top 10 busiest US airports by total passenger traffic were pre-set as generator points. Also, as previously shown in Fig. 9, Alaska has its trend to form its own cluster, so two additional cases were investigated, where Ted Stevens Anchorage International Airport (ANC) was included as an additional generator point besides the top 5 and top 10 busiest airports (Fig. 10 and Supplementary Fig. 5). The 10 busiest airports based on total passenger count in 2018 in the United States are as follows: Hartsfield–Jackson Atlanta International Airport (ATL), Los Angeles International Airport (LAX), O’Hare International Airport (ORD), Dallas/Forth Worth International Airport (DFW), Denver International Airport (DEN), John F. Kennedy International Airport (JFK), San Francisco International Airport (SFO), Seattle–Tacoma International Airport (SEA), McCarren International Airport (LAS) and Orlando International Airport (MCO). This list is based on annual passenger traffic figures published for 2018 by each airport authority. From all four figures (Fig. 10 and Supplementary Figs. 1, 2, 3) one can draw two major conclusions: (1) the optimal clustering found by the Voronoi algorithm is in good agreement with the catchment area of the biggest airports; (2) the introduced local relative density measure is proportional with the real life data of actual annual traffic of each airport.

### Evaluation against other algorithms on artificial benchmark networks

Benchmark networks used in the current study were created based on the popular LFR algorithm^23,43^, which generates a random network with a predefined community structure. Here we used the original implementation for directed graphs, written by the authors of the algorithm^43^. The LFR benchmark generator requires specifying a so-called mixing parameter $\mu \in (0, 1)$, which controls how well the generated communities are separated from each other, in other words, how easy it is to distinguish between the clusters. Specifically, a fraction $1-\mu$ of links is contained within communities. As the value of the mixing parameter is increased, the clusters are less separated, and it becomes harder to detect them accurately. We generated benchmark sets corresponding to the full range of mixing parameter values $\mu = 0.1, 0.2, \dots , 0.9$. The other parameters of the LFR benchmark model were fixed as $N=1000$ nodes, average degree ${\bar{k}} = 100$, maximum in-degree $k_\text {max} = 300$.

Although the benchmark network generator software^43^ is capable of producing link weights, we only used it to create binary (unweighted) networks and sampled the edge weights separately. This way we were able to study how different weight distributions influence the performance of the Voronoi algorithm. Two different types of edge weight distributions were considered: (1) Truncated normal distribution with probability density $p(w) \sim e^{-\frac{1}{2} \left( \frac{w-m}{\sigma }\right) ^2}$, $w \in (0, \infty )$; and (2) power distribution with probability density $p(w) \sim \alpha \, w^{\alpha - 1}$, $w \in (0, 1]$ where $0 < \alpha \le 1$. Since the power distribution assigns very high probability to tiny values which may become rounded to zero during floating point computations, its support was truncated to $w \ge w_\text {min} = 0.01$. The expected value of this truncated power distribution is $m = \frac{\alpha }{1+\alpha } \frac{w_\text {min}^{\alpha +1} - 1}{w_\text {min}^\alpha }$.

The steps for generating the random weighted and directed benchmark networks are the following: (1) generate a binary directed network with a specific mixing parameter $\mu$; (2) choose the type of edge weight distribution (normal or power); (3) sample the edge weights, choosing different distribution parameters for inter- and intra-community edges, so that the average weight of links within communities would be no smaller than that of links connecting distinct communities, $m_\text {intra} > m_\text {inter}$.

**Figure 11:**
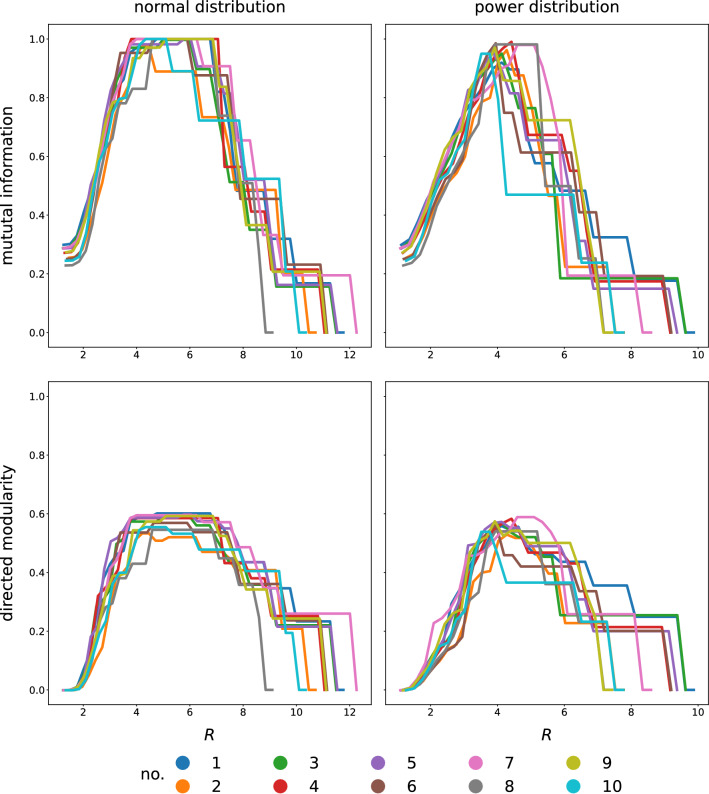
Mutual information (MI) between the clustering obtained by the Voronoi algorithm and the ground-truth clustering encoded in the benchmark networks as function of radius *R* (upper row) and directed Newman modularity as a function of radius *R* (lower row) in case of normal (left column) and power (right column) weight distributions of the same 10 (see legend) networks with $N=1000$, $k = 100$ and mixing parameter $\mu =0.3$. The base network is the same for both distribution types, only the weights differ according to the distribution parameters chosen to have the same distribution mean in both cases of normal and power distributions: $m_\text {intra}=0.58, m_\text {inter}=0.42$ for normal, while $\alpha _\text {intra}=0.7, \alpha _\text {inter}=0.3$ for power distributions.

Since weight values represent the strength of each link in this case, we used the $f(w_{ij})=1/w_{ij}$ transformation when calculating the length of links. Figure 11 shows the mutual information—a measure characterizing the similarity of two different partitionings of the same network^44^—between the ground truth community structure and the detected one, as well as the directed modularity values, as a function of the *R* parameter in 10 networks with $N=1000$ nodes, ${\overline{k}}=100$ average degree ($k_{max}=300$) and mixing parameter $\mu =0.3$. Most curves show a plateau around their maximum, indicating that the optimal clustering can be achieved for a range of *R* values.

**Figure 12:**
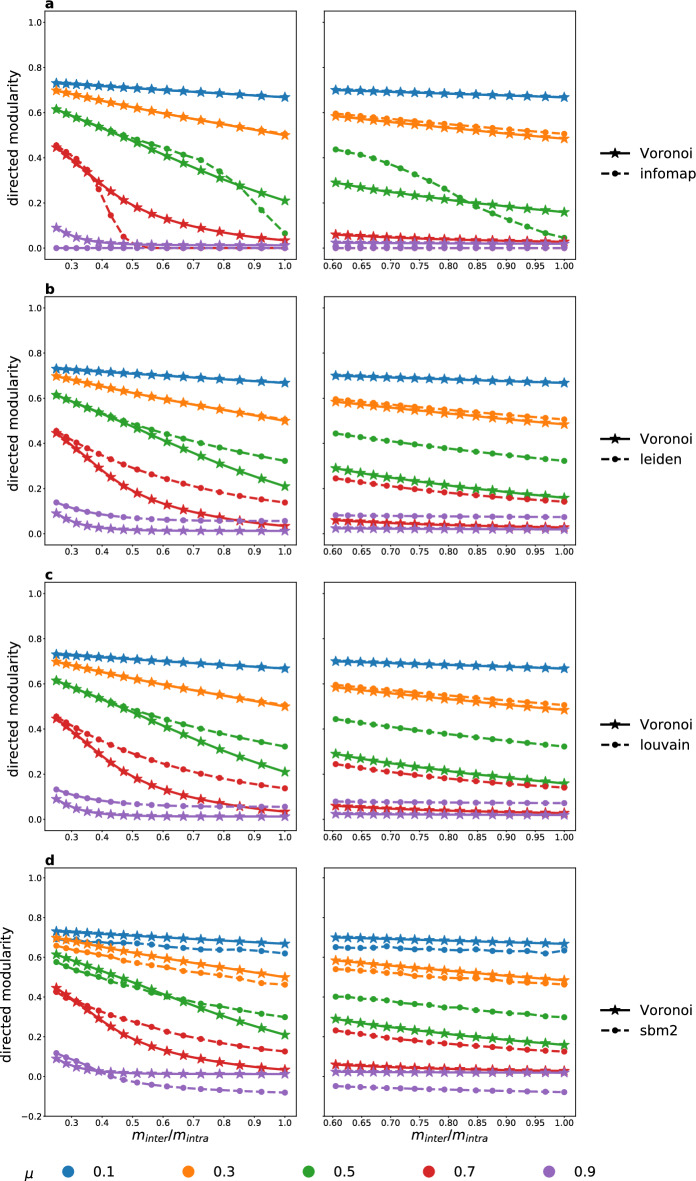
Performance comparison of the Voronoi algorithm (solid line, stars) and (**a**) Infomap, (**b**) Leidenalg, (**c**) Louvain algorithm and (**d**) SBM (all represented by dashed line, dots) using a total of $288\,000$ benchmark networks. The modularity of the detected communities is shown as a function of the weight ratio between inter- and intra-community links. Curves of different colors correspond to LFR benchmark networks generated with different mixing parameters ($\mu =0.1, 0.3,\dots , 0.9$). All benchmark networks have $N=1000$ nodes and mean degree ${\bar{k}} = 100$. The parameters of the link weight distributions were chosen for both, normal and power distributions, as follows: $(m_\text {inter}, m_\text {intra}) = (0.20, 0.80), (0.22, 0.78) \dots , (0.50, 0.50)$. Each data point is averaged over 1000 LFR networks.

Figure 12 shows a performance comparison of the Voronoi algorithm with Infomap^7^, the Louvain clustering algorithm^3^, the Leiden algorithm^11,13^ and fitting to a Stochastic Block Model^10^, which are the most popular community detection methods used with weighted and directed networks. Each curve shows the modularity as function of the $m_\text {inter}/m_\text {intra}$ inter-intra average weight ratio for benchmark networks created with a given mixing parameter. Simulations were run for all $\mu =0.1, 0.2, \dots , 0.9$ values, using a total of $288\,000$ benchmark networks. In Fig. 12 we show only the curves for $\mu =0.1, 0.3, 0.5, 0.7, 0.9$ to increase visibility. As the mixing parameter increases, communities become less separated and harder to detect. The inter- and intra-community weight distributions are varied from more distinct to more similar in the figure panels from left to right, with the horizontal axis showing the ratio of the average inter- and intra-community weights. The smaller this ratio, the easier it is to detect communities accurately. We observe that while all community detection methods perform well on easy benchmark instances, they differ in how their performance decreases as the problem gets more difficult. In the Supplementary Information we also plot the mutual information (Fig. S6) and accuracy (Fig. S7) to compare the community structure with the ground truth.

Infomap exhibits an almost binary behaviour: it either recovers the ground truth community structure nearly perfectly, or it fails to find any communities at all. Closer examination reveals that when this happens, Infomap either returns a single community, or places each vertex in its own size-1 community. In contrast, the performance of the Voronoi algorithm decreases gradually, and it still manages to recover a reasonably accurate community structure on difficult problem instances (Fig. 12a). The Leiden and Louvain algorithms exhibit an almost identical behaviour, because they both optimize modularity. Given that here we plot modularity, it is expected that these two algorithms perform the best. Indeed, we can see in Fig. 12b,c that they outperform our algorithm in the sense of providing higher modularities. The stochastic block modelling performs slightly worse than the Voronoi algorithm on small and large mixing parameters. There is a medium interval, however, especially in case of power-law weight distributions, where it provides higher modularity values than our algorithm.

**Figure 13:**
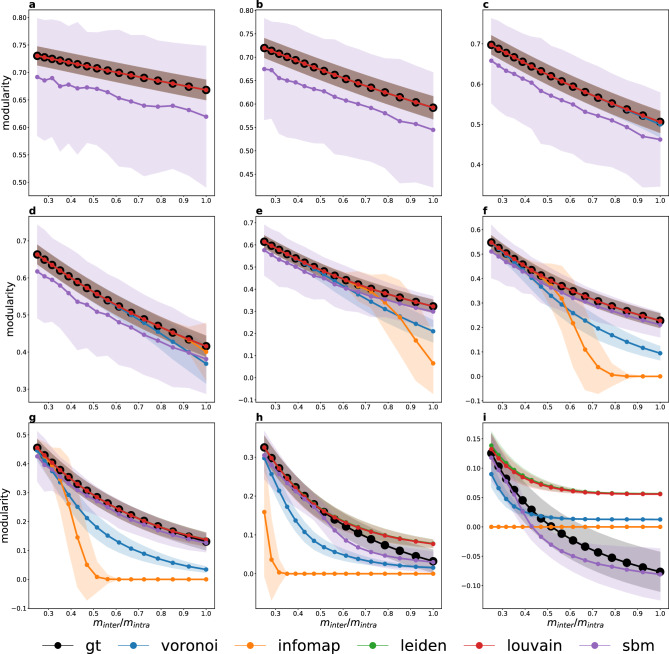
Comparing the average modularity obtained by different clustering methods (see legend) to the ground truth modularity values using a total of $288\,000$ benchmark networks. The modularity of the detected communities is shown as a function of the weight ratio between inter- and intra-community links. Curves of different colors correspond to different clustering methods (see legend), while different panels represent LFR benchmark networks generated with different mixing parameters (**a**) $\mu =0.1, \dots , 0.9$). All benchmark networks have $N=1000$ nodes and mean degree ${\bar{k}} = 100$. The parameters of the link weight distributions were chosen for normal distributions, as follows: $(m_\text {inter}, m_\text {intra}) = (0.20, 0.80), (0.22, 0.78) \dots , (0.50, 0.50)$. Each data point is averaged over 1000 LFR networks.

In order to compare more easily all algorithms with the ground truth in Fig. 13 we plot the same curves as in the first column of Fig. 12, but grouped by the mixing parameter values separately on each subfigure. The modularity value in the ground truth is shown in black. We can see that in the last two subfigures (Fig. 13h,i) the algorithms are already capable of finding higher modularity values than the ground truth, showing the importance of weights that can overwrite the clustering encoded by the simple topology (the ground truth will not be necessarily the optimal clustering anymore).

**Figure 14:**
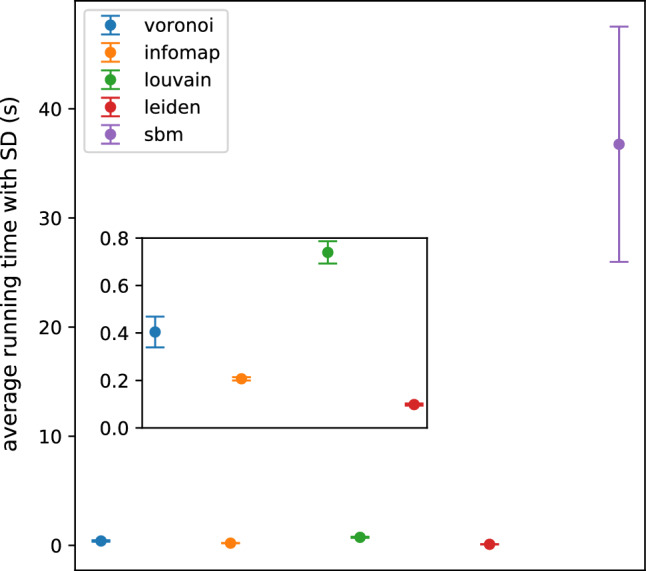
The average running time with the error bar indicating the SD calculated on 100 benchmark networks with $N=1000$ nodes, average degree ${\overline{k}}=100$, mixing parameter $\mu =0.3$, with power law weight distribution having $\alpha _\text {intra}=0.6$ for intra-community, respectively $\alpha _\text {inter}=0.4$ for inter-community edge weights. The Voronoi (blue), Infomap (orange), Louvain (green), Leiden (red) and SBM (purple) algorithms are compared.

A speed performance comparison was conducted between the algorithms (Fig. 14) on an Apple MacBook Pro (16 inch, 2019) laptop using 100 randomly generated benchmark networks with $N=1000$ nodes, average degree ${\overline{k}}=100$, mixing parameter $\mu =0.3$, with power law weight distribution having $\alpha _\text {intra}=0.6$ for intra-community, respectively $\alpha _\text {inter}=0.4$ for inter-community edge weights. We can see that our algorithm performs in the same range as the fastest algorithms.

### Robustness of the generator point selection

To demonstrate the efficiency and robustness of the generator point selection, we investigated Voronoi partitions obtained after a random rearrangement of the generator points. We consider two types of rearrangements. In the first approach, an initial Voronoi clustering is constructed using generator points selected in the usual manner, based on high local relative density, and finding the optimal *R* parameter value. Then each generator point is replaced by a node selected randomly from within its cluster. We refer to this as *intra-community* randomization. In the second approach, the same number of generator points are sampled uniformly at random (without replacement) from the set of all nodes. We call this *uniform* randomization. Finally, a new Voronoi clustering is obtained from the rearranged generator points.

**Figure 15:**
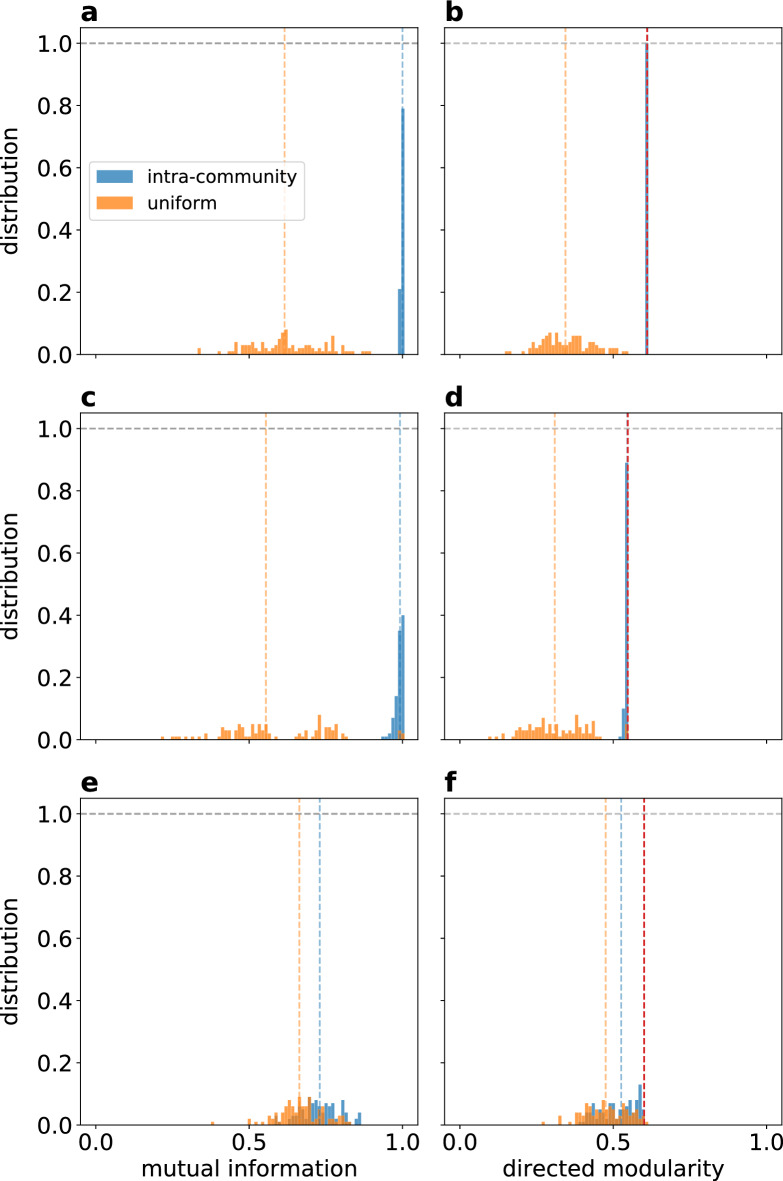
Histogram of mutual information (**a**, **c**, **e**) and directed modularity (**b**, **d**, **f**) values for 100 randomized generator point set in case of: (**a**, **b**) a benchmark network with higher ground truth directed modularity value ($Q=0.610638$); (**c**, **d**) a benchmark network with lower ground truth directed modularity value ($Q=0.547427$) and (**e**, **f**) the cortical functional network of the macaque monkey. Both random benchmark networks have $N=1000$ nodes, an average degree ${\overline{k}}=100$, mixing parameter $\mu =0.3$ and the link weights follow a power distribution with $\alpha _\text {intra}=0.7$ and $\alpha _\text {inter}=0.3$ for intra- and inter-community links, respectively. Blue bars represent the intra-community randomization of the generator points, while the orange ones the uniform randomization. The vertical blue and orange dashed lines show the median of the distribution, while the vertical red dashed line on the directed modularity plots represents the directed modularity value of the ground truth clustering in case of the benchmark networks and the optimal state modularity obtained by the Voronoi algorithm in case of the cortical network of the macaque.

For this experiment, we used two LFR benchmark networks as well as the macaque brain connectivity network. The two benchmark networks were both generated using the same LFR parameters ($N=1000$ nodes, average degree ${\overline{k}}=100$ and mixing parameter $\mu =0.3$) and the same weight distributions (power law distributions with exponents $\alpha _\text {intra}=0.7$ for intra-community and $\alpha _\text {inter}=0.3$ for inter-community links). The two benchmark networks were specifically selected from a large randomly generated set so that their ground truth community structures would have very different modularity values ($Q=0.610638$ in Fig. 15a,b and $Q=0.54742$ in Fig. 15c,d).

Each type of randomization was repeated 100 times on these three networks, and the obtained clusterings were evaluated using the modularity and mutual information metrics. In case of the benchmark networks, the ground truth community structure was used as the baseline of the evaluation. Since no ground truth is available for the macaque brain network, the optimal Voronoi clustering was used in its place. The obtained results are shown in Fig. 15, where light blue colors represent the results obtained after intra-community randomization, while the orange shows the results for uniform randomization. Red dashed lines indicate the baseline modularity.

With both the high and low modularity benchmark networks, the Voronoi algorithm, in its original form, was able to recover the ground truth clustering exactly, as indicated by a mutual information value of 1. Intra-community randomization had very little effect on the result (blue). This demonstrates that when precisely one generator point is placed into each community, the Voronoi approach is accurate in recovering the true community structure, regardless of the specific location of the generators. The local relative density-based generator selection method is very effective in achieving this one generator per community arrangement. In comparison, a random placement of generators leads to a significantly worse result (orange).

In case of the macaque brain network, the obtained clustering is more sensitive to intra-community randomization. However, it must be noted that this 29-node network is much smaller than the 1000-node benchmark graphs, more dense, so topologically less modular (its modular nature is hidden more in the link weights) therefore large fluctuations are to be expected. Even so, intra-community randomization produces clusterings with clearly higher modularity than uniform randomization, which supports the original choice of generator points, based on local relative density. Our original method shown as the baseline (red dashed line) provides almost the maximal modularity achieved on this network.

## Discussion

In this paper we presented a computationally efficient method for detecting community structure in directed and weighted networks. An advantage of our algorithm is that it can make direct use of edge weights which represent additive *distances*, i.e. data in which larger weights indicate weaker connections. Most other community detection methods, including all of those that maximize modularity explicitly, interpret weights as *connection strengths*, and thus require transforming distance data before they can be used. This transformation is often done in an ad-hoc manner. In contrast to such methods, Voronoi community detection provides a principled way to work with distance-weighted networks. This feature is similar to how some network metrics, such as betweenness centrality, employ distances, while others, such as PageRank, use connection strengths.

While Voronoi community detection is a natural fit for distance-weighted data, it can also work with weights of different types after applying a suitable weight transformation. The most appropriate transformation depends on the specific use case. We discuss several possible approaches to this, which covers many different types of networks.

Regarding directions, we have chosen to use the outgoing paths (defining the region with radius *R* with shortest paths outgoing from a node) when choosing generator nodes, and also when deciding the community of each node they belong to (calculating the shortest path starting from the node that needs to be assigned to a cluster to the different generator nodes). This choice is motivated by networks that carry some kind of flow, for example information flow in brain networks. One may intuitively think of nodes with strong incoming connections as points of attraction, and the Voronoi cell of a generator point as its sphere of attraction. However, the same method may also be used with incoming paths, or even compare the community structure obtained in the two different cases.

The *R* controls the minimal distance between generator nodes (no pair of generator nodes can be closer than *R*), and through this it almost directly controls the diameter of the largest few clusters. Nevertheless, it does not force all clusters to have similar diameters. There are many data sets where there are just a few large relevant communities and many small ones, the number of small communities depends a lot on the precision of data (see for example the air transportation network, or the friendship networks). If one knows from the data the meaning of distances defined in the network, one can define a relatively appropriate *R* parameter that can provide meaningful results. Here we usually mapped the whole interval of *R*, in order to study in details the properties of the algorithm. As we have shown in case of benchmark networks, there is typically a range of *R* radius values which yield the optimal clustering, which helps the time performance of the algorithm when searching for the optimal *R* value. The most computationally expensive part of the algorithm is calculating shortest path lengths in the graph. This can be realized with Dijkstra’s algorithm^32^ performed on each generator node leading to a computational complexity ${\mathcal {O}}(\log |S| |E|\log |V|)$.

We contributed an implementation of the Voronoi community detection algorithm (igraph_community_voronoi()) and its building blocks (igraph_ecc(), igraph_voronoi()) to the *igraph*^45,46^ open-source network analysis software, making the method easily available to practitioners of network analysis.

### Supplementary Information

Supplementary Information.

## Data Availability

The codes for generating weighted benchmark datasets and comparing our method with other well-known algorithms are available at: https://github.com/molnarb14/directed-weighted-community-detection-voronoi. The datasets used in the mouse^18^ and macaque^19^ brain study are available with the respective publications. The transportation networks used in the current study are publicly available from the https://www.transtats.bts.gov/ website. The two social networks, as well as the Austrian migration network, are downloadable from the Netzschleuder database at https://networks.skewed.de/about.
