# Supplementary material for: Community detection in directed weighted networks using Voronoi partitioning
Source: Sci Rep. 2024 Apr 7;14:8124. doi: 10.1038/s41598-024-58624-4 (PMC10998900; doi:10.1038/s41598-024-58624-4)
Supplement: Supplementary file 1 — Supplementary Information. [file 41598_2024_58624_MOESM1_ESM.pdf]

Supplementary Information for

Community Detection in Directed Weighted Networks using Voronoi Partitioning

Molnár et al.

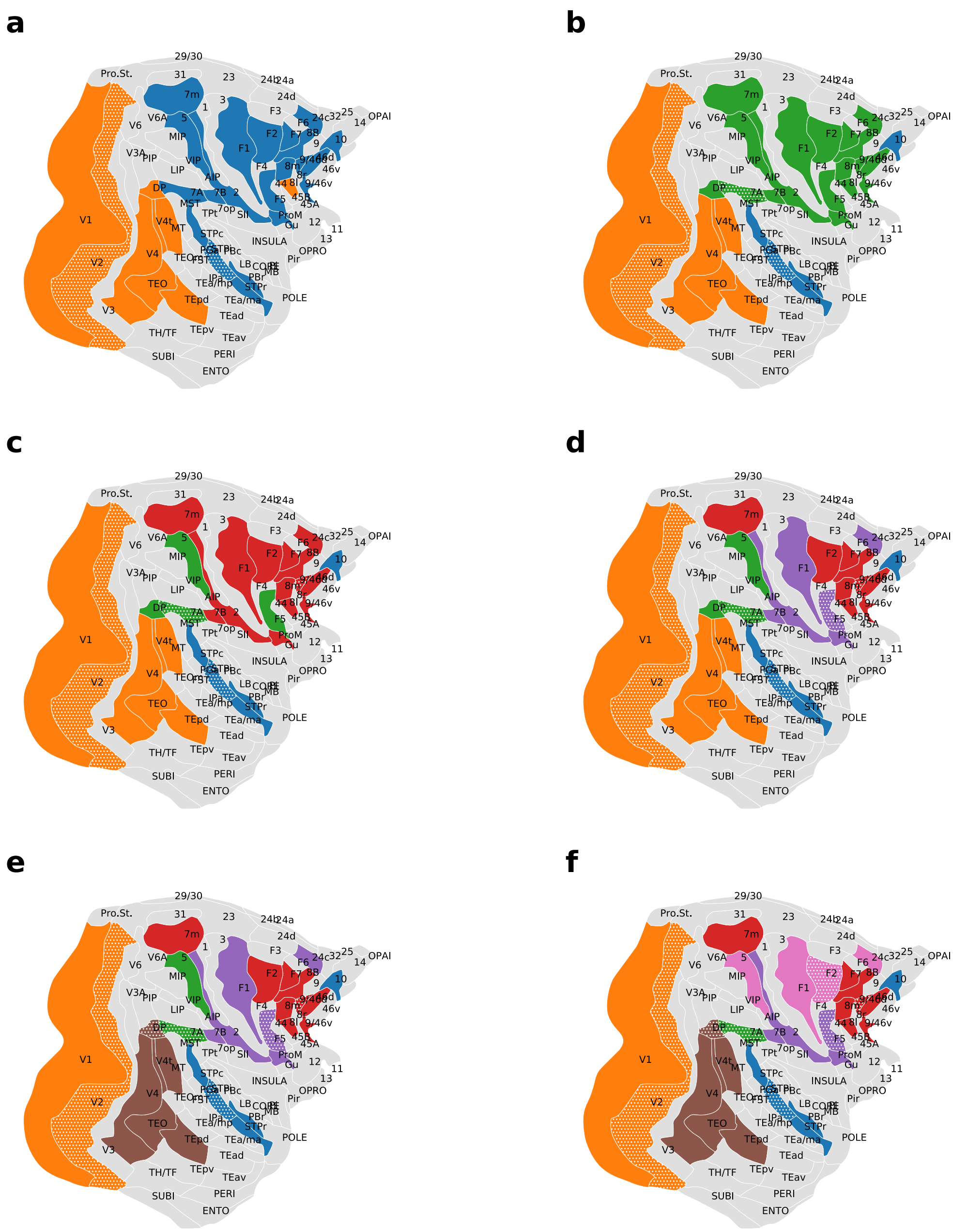

Supplementary Figure 1. Clustering of cortical brain areas in the macaque monkey based on their structural connectivity. Colors represent different clusters, while the generator nodes are marked with a dotted pattern. Connectivity data was unavailable for the light gray areas; thus, these were excluded from the clustering. Panels a)-f) show partitioning into 2–7 clusters, as obtained with the Voronoi algorithm. The highest modularity is achieved with 7 clusters.

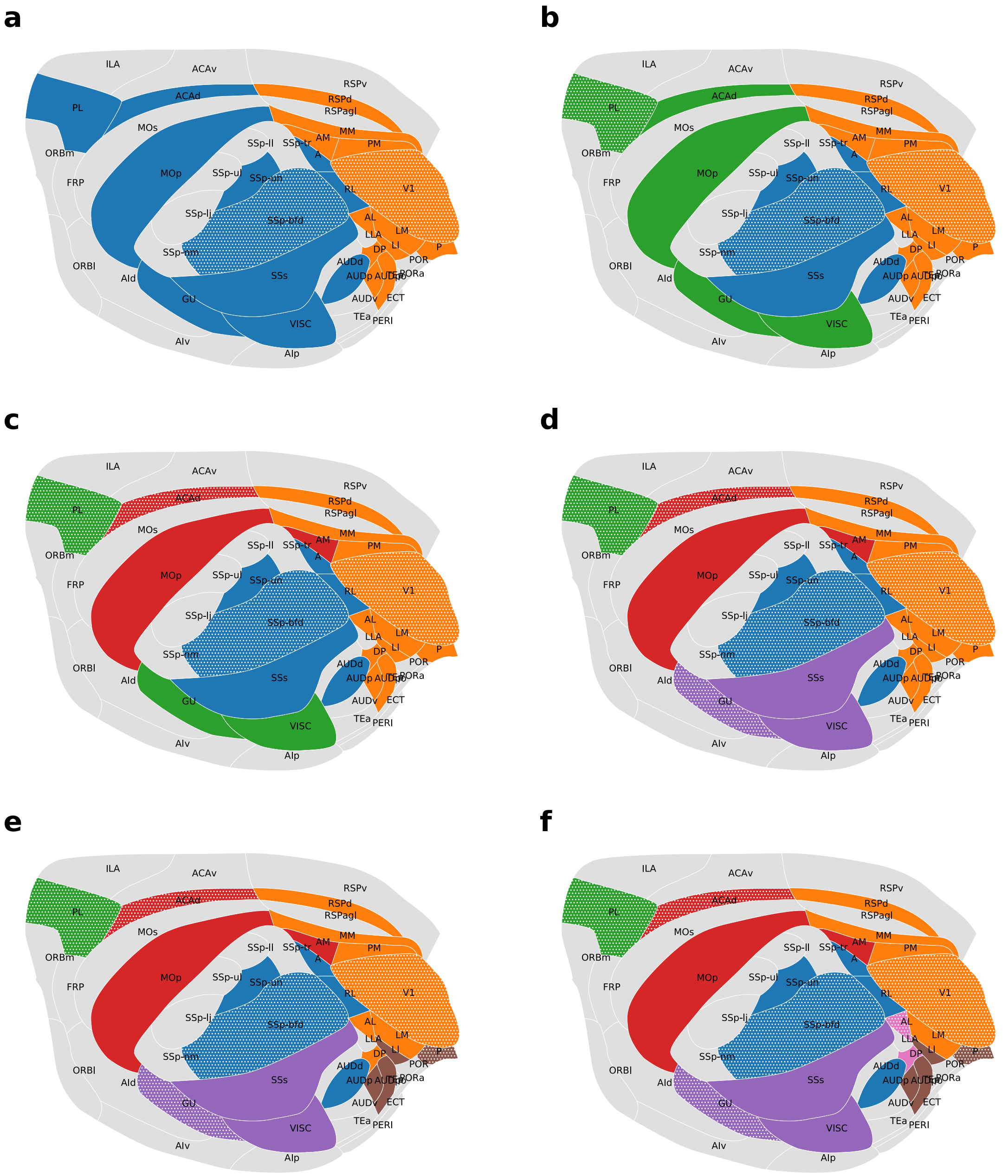

Supplementary Figure 2. Clustering of the brain network in case of the mouse. Colors represent different clusters; the patterns show the generator nodes of clusters. The light gray functional areas are not included in the clustering due to the missing information about these areas. The Voronoi algorithm provided a fixed number of clusters, as follows: a) two; b) three; c) four; d) five; e) six; respectively f) seven clusters. In case of the mouse the optimal clustering is obtained with 2 clusters.

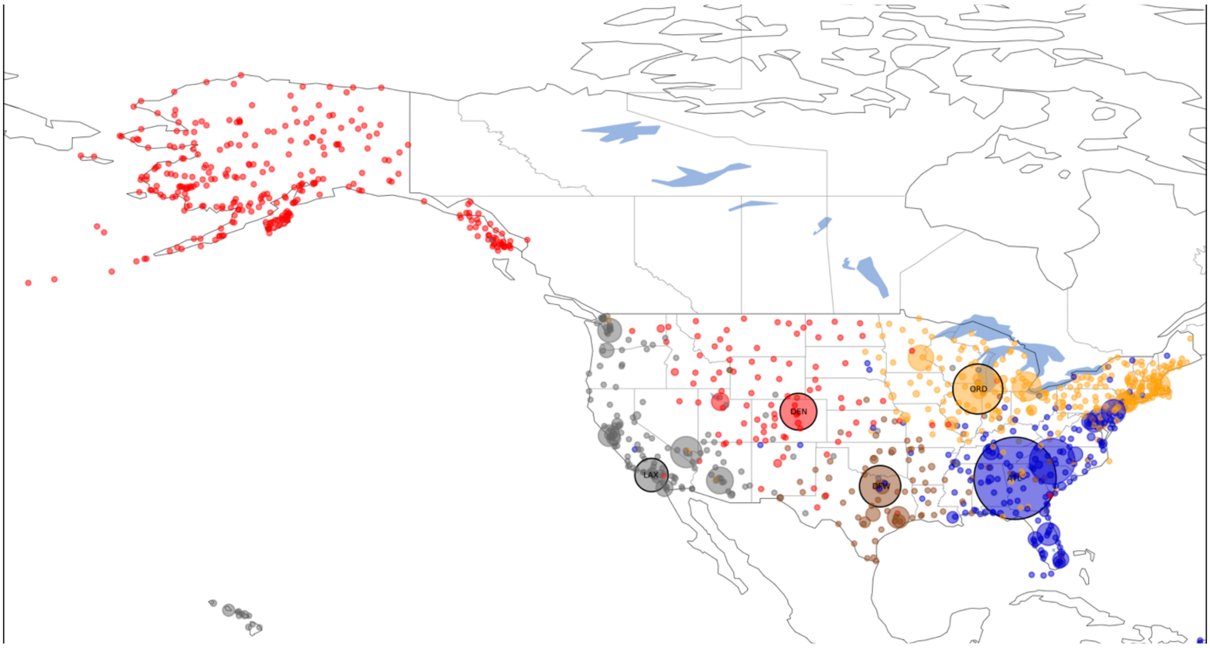

Supplementary Figure 3. Clustering of the air passenger transportation network of the United States using the Voronoi algorithm using the Top 5 busiest airports (ATL, LAX, ORD, DFW, DEN) as generator points. The dots represent the locations of airports on the map, their size is proportional to the local density. Colors represent the different clusters; the black border indicates the generator nodes. Only generator point airports display the IATA airport code.

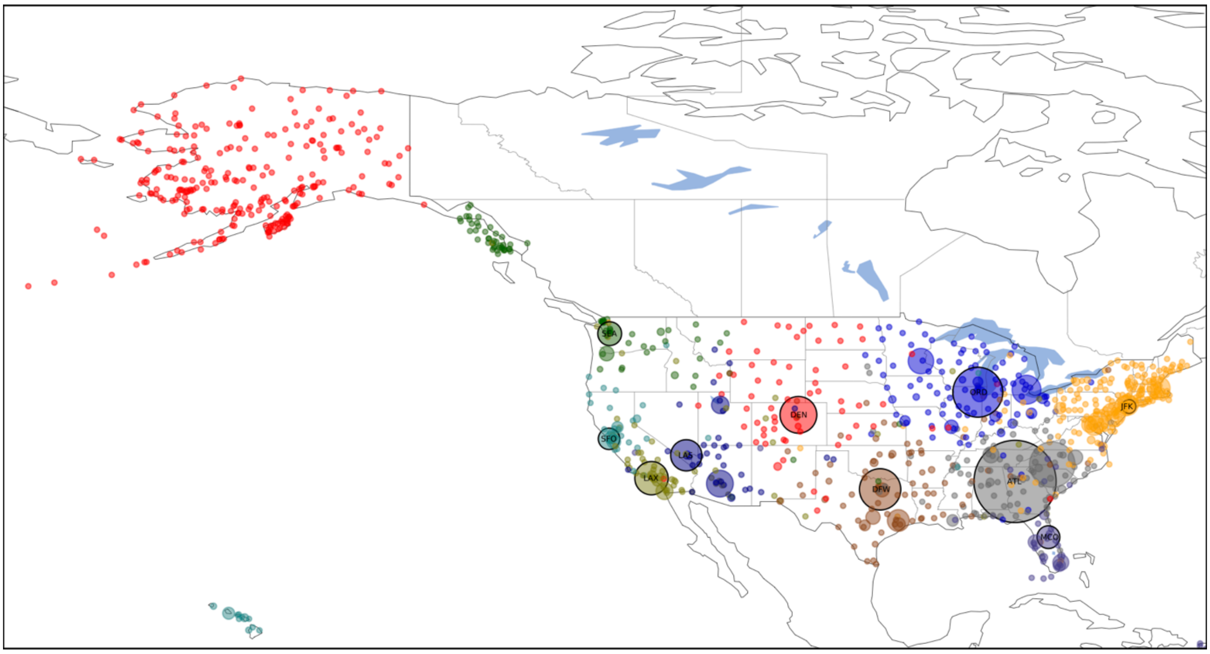

Supplementary Figure 4. Clustering of the air passenger transportation network of the United States using the Voronoi algorithm using the Top 10 busiest airports (ATL, LAX, ORD, DFW, DEN, JFK, SFO, SEA, LAS, MCO) as generator points. The dots represent the locations of airports on the map, their size is proportional to the local density. Colors represent the different clusters; the black border indicates the generator nodes. Only generator point airports display the IATA airport code.

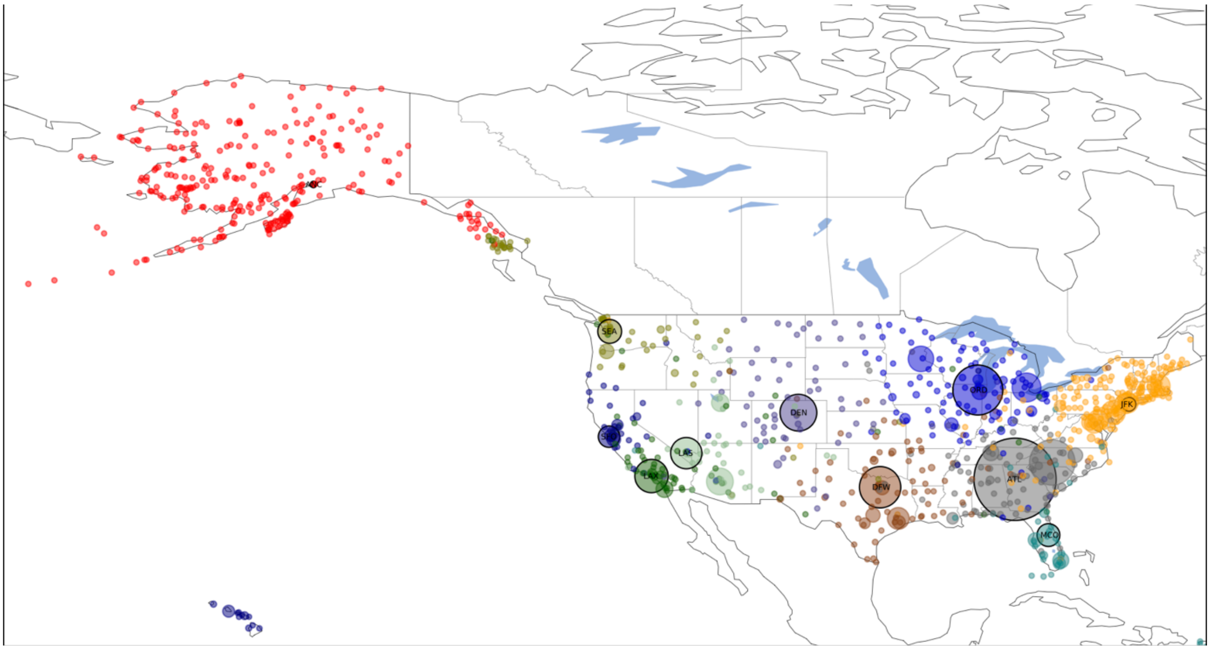

Supplementary Figure 5. Clustering of the air passenger transportation network of the United States using the Voronoi algorithm using the Top 10 busiest airports (ATL, LAX, ORD, DFW, DEN, JFK, SFO, SEA, LAS, MCO) and ANC as generator points. The dots represent the locations of airports on the map, their size is proportional to the local density. Colors represent the different clusters; the black border indicates the generator nodes. Only generator point airports display the IATA airport code.

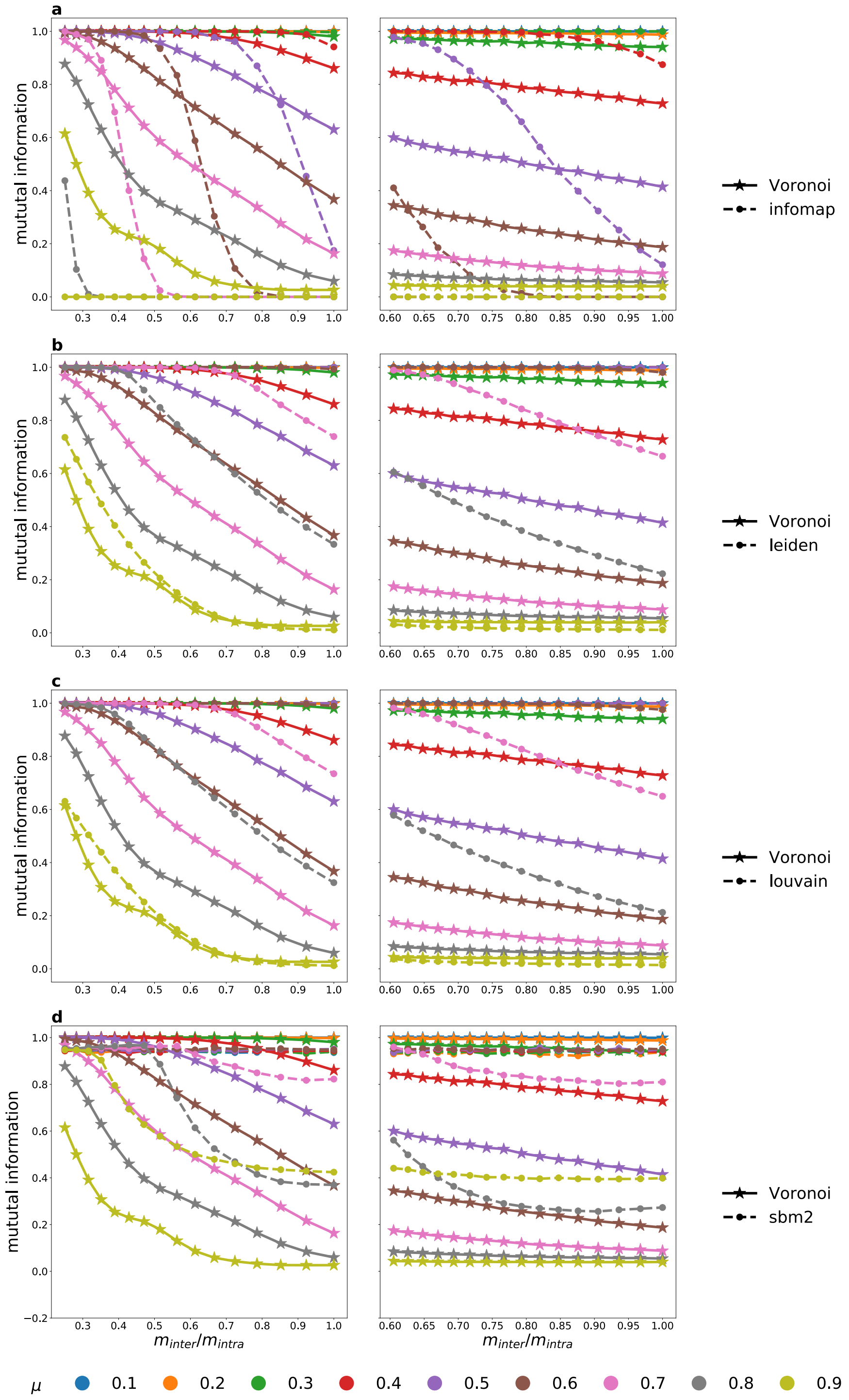

Supplementary Figure 6. Performance comparison of the Voronoi algorithm (solid line, stars) and a) Infomap, b) Leidenalg, c) Louvain algorithm and d) SBM (all represented by dashed line, dots) using a total of 288 000 benchmark networks. The mutual information with the ground truth clustering is shown as a function of the weight ratio between inter- and intra-community links. Curves of different colors correspond to LFR benchmark networks generated with different mixing parameters (m = 0.1, 0.2, …, 0.9). All benchmark networks have $N = 1000$ nodes and mean degree $\bar{k} = 100$. The parameters of the link weight distributions were chosen for both, normal and power distributions, as follows: $(m_{inter}; m_{intra}) = (0.20, 0.80), (0.22, 0.78), \dots, (0.50, 0.50)$. Each data point is averaged over 1000 LFR networks.

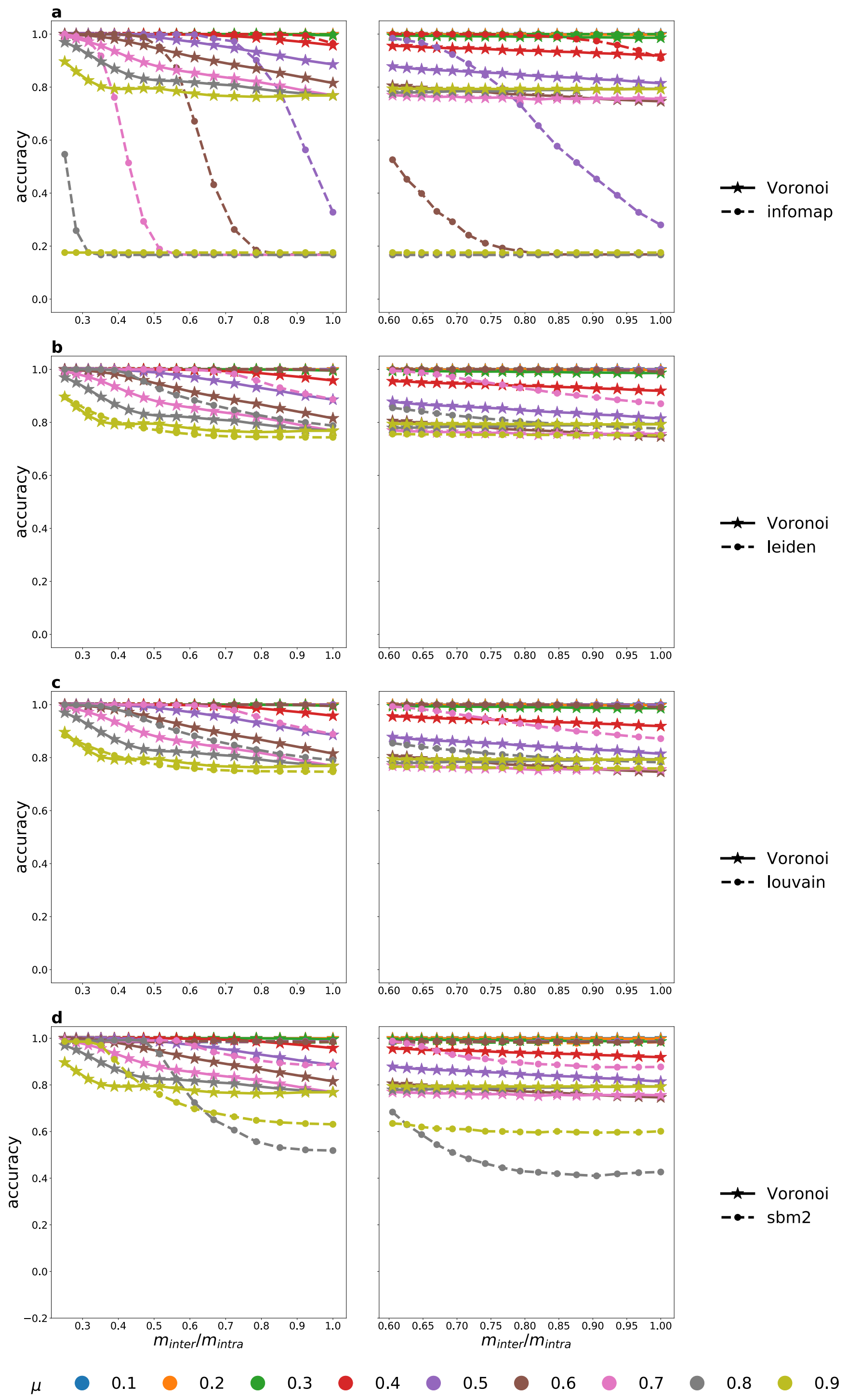

Supplementary Figure 7. Performance comparison of the Voronoi algorithm (solid line, stars) and a) Infomap, b) Leidenalg, c) Louvain algorithm and d) SBM (all represented by dashed line, dots) using a total of 288 000 benchmark networks. The accuracy of the obtained clustering is calculated compared to the ground truth clustering and shown as a function of the weight ratio between inter- and intra-community links. Curves of different colors correspond to LFR benchmark networks generated with different mixing parameters (m = 0.1, 0.2, …, 0.9). All benchmark networks have $N = 1000$ nodes and mean degree $\bar{k} = 100$. The parameters of the link weight distributions were chosen for both, normal and power distributions, as follows: $(m_{inter}; m_{intra}) = (0.20, 0.80), (0.22, 0.78), \ldots, (0.50, 0.50)$. Each data point is averaged over 1000 LFR networks.

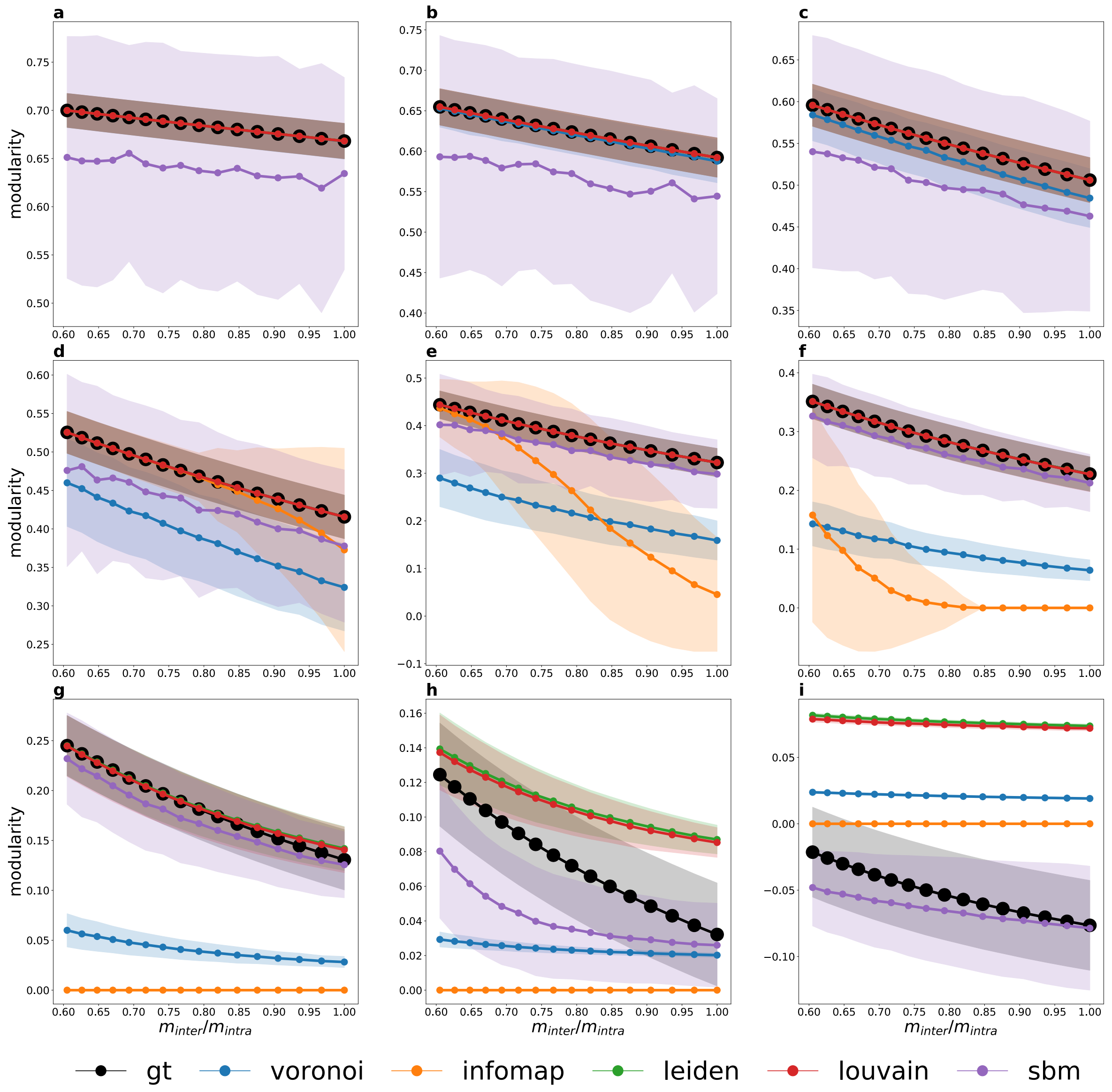

Supplementary Figure 8. Comparing the average modularity obtained by different clustering methods (see legend) to the ground truth modularity values using a total of 288 000 benchmark networks. The modularity of the detected communities is shown as a function of the weight ratio between inter- and intra-community links. Curves of different colors correspond to different clustering methods (see legend), while different panels represent LFR benchmark networks generated with different mixing parameters a) m = 0.1, …, i) 0.9. All benchmark networks have $N = 1000$ nodes and mean degree $\bar{k} = 100$. The parameters of the link weight distributions were chosen for normal distributions, as follows: $(m_{inter}; m_{intra}) = (0.20, 0.80), (0.22, 0.78), \dots, (0.50, 0.50)$. Each data point is averaged over 1000 LFR networks.
